# Mobile App Promoting Resilience in Stress Management for Adolescents and Young Adults With Cancer: Protocol for a Pilot Randomized Controlled Trial

**DOI:** 10.2196/57950

**Published:** 2024-07-30

**Authors:** Nancy Lau, Tonya M Palermo, Chuan Zhou, Isabel Badillo, Shannon Hong, Homer Aalfs, Joyce P Yi-Frazier, Elizabeth McCauley, Eric J Chow, Bryan J Weiner, Dror Ben-Zeev, Abby R Rosenberg

**Affiliations:** 1 Center for Child Health, Behavior and Development Seattle Children’s Research Institute Seattle, WA United States; 2 Department of Psychiatry & Behavioral Sciences University of Washington School of Medicine Seattle, WA United States; 3 Department of Anesthesiology & Pain Medicine University of Washington School of Medicine Seattle, WA United States; 4 Department of Pediatrics University of Washington School of Medicine Seattle, WA United States; 5 Department of Psychosocial Oncology and Palliative Care Dana-Farber Cancer Institute Boston, MA United States; 6 Clinical Research and Public Health Sciences Divisions Fred Hutch Cancer Center Seattle, WA United States; 7 Department of Global Health University of Washington Seattle, WA United States; 8 Department of Pediatrics Boston Children’s Hospital Boston, MA United States; 9 Department of Pediatrics Harvard Medical School Boston, MA United States

**Keywords:** adolescents, young adult, cancer, mHealth, psychosocial intervention, stress management, coping, resilience, health-related quality of life, randomized controlled trial, mobile phone

## Abstract

**Background:**

Adolescents and young adults (AYAs) with cancer are at risk of poor psychosocial outcomes. AYAs grew up with the internet and digital technology, and mobile Health (mHealth) psychosocial interventions have the potential to overcome care access barriers.

**Objective:**

This pilot randomized controlled trial (RCT) aimed to establish the feasibility, acceptability, and preliminary efficacy of a fully automated mobile app version of the Promoting Resilience in Stress Management intervention (mPRISM). Promoting Resilience in Stress Management is an evidence-based intervention developed in collaboration with AYAs, based on stress and coping theory, resilience theory, and evidence-based coping strategies. We hypothesized that mPRISM would be feasible, acceptable, and appropriate.

**Methods:**

This is a parallel, 2-arm, single-site pilot RCT with a waitlist control design. The study will recruit 80 AYAs with cancer from a clinic. Eligible AYAs are aged 12 to 25 years, within 12 months of a new cancer diagnosis, receiving chemotherapy or radiation therapy, speak, read, or write in English, and are cognitively able to participate in study procedures. Recruitment by clinical research coordinators will occur remotely by phone, video, or text. Participants will be randomized to psychosocial usual care (UC) alone or UC plus mPRISM for an 8-week intervention period, and will remain unblinded to study condition. Enrolled participants will complete surveys at baseline before randomization, 8 weeks, and 3-month follow-up. Using a waitlist design, the UC arm will receive mPRISM upon completion of 3-month follow-up surveys. Those in the UC arm will complete 2 additional measurement points at immediate posttreatment and 3 months later. The primary outcomes of interest are feasibility, defined as ≥60% enrollment and ≥70% retention (ie, percentage of participants who completed the study), and “feasibility, acceptability, and appropriateness” as defined by cut-off scores ≥4/5 on 3 brief validated implementation outcome measures (feasibility of implementation measure, acceptability of intervention measure [AIM], intervention appropriateness measure [IAM]). We will apply top-box scoring for the implementation measures. Exploratory outcomes of interest include patient-reported health-related quality of life, resilience, distress, anxiety, depression, pain, and sleep. We will conduct an intention-to-treat analysis to compare the outcomes of the mPRISM arm versus the control arm with covariate-adjusted regression models. We will summarize individual digital usage metrics using descriptive statistics.

**Results:**

Since September 2023, we have enrolled 20 participants and recruitment is ongoing.

**Conclusions:**

Although our previous work suggests AYAs with cancer are interested in mHealth psychosocial interventions, such interventions have not yet been sufficiently evaluated or implemented among AYA oncology patients. mPRISM may serve as a potential mHealth intervention to fill this gap. In this study, we will test the feasibility, acceptability, and preliminary efficacy of mPRISM. This work will inform future larger-scale RCTs powered for efficacy outcomes.

**Trial Registration:**

ClinicalTrials.gov NCT05842902; https://clinicaltrials.gov/study/NCT05842902

**International Registered Report Identifier (IRRID):**

DERR1-10.2196/57950

## Introduction

Pediatric adolescents and young adults (AYAs), 12-25 years old, with cancer experience unique developmental and psychosocial needs because cancer disrupts typical developmental experiences and life transitions including the pursuit of educational and vocational goals and the establishment of social, personal, and sexual identity [1]. AYAs with cancer experience poor psychosocial outcomes including social isolation, behavioral adjustment problems, depression, anxiety, and stress [2,3]. In addition, quality of life (QoL) and psychosocial outcomes among AYA cancer survivors are inferior to their younger pediatric and older adult counterparts [4]. Such sequelae are further associated with negative long-term survivorship outcomes such as low educational attainment, unemployment, and financial dependence [5]. Elevated psychological distress in AYA cancer survivors is associated with medical costs of US $4415 per year [6].

The Promoting Resilience in Stress Management (PRISM) program is an evidence-based resilience intervention designed for and in collaboration with AYAs [7-13]. PRISM is a brief, manualized, evidence-based intervention delivered one-to-one in person by layperson coaches. PRISM was developed based on iterative research within the AYA and pediatric oncology population, stress and coping theory, resilience theory, and evidence-based cognitive-behavioral and mindfulness interventions [7-13]. Previous studies suggest in-person delivery of PRISM is feasible and valuable to AYA patients and parents [11,12]. In a randomized controlled trial (RCT) of AYAs with cancer, PRISM was associated with improved self-reported resilience, distress, and health-related quality of life (HRQoL), with moderate effect sizes [14,15]. However, despite these promising effects, major barriers limit the dissemination and reach of in-person interventions such as PRISM, including geographic proximity, transportation, limited trained personnel, and treatment costs.

Skills-based psychosocial interventions are increasingly deployed through mobile Health (mHealth) platforms [16,17] and show promise in early research in youth with chronic illnesses [18]. Smartphones have become ubiquitous [19], irrespective of gender, race and ethnicity, and socioeconomic status [20]. The implementation of mHealth-based care delivery has the potential to improve access to evidence-based treatment and reduce health disparities; for example, 73% of families living below the poverty line in the United States have one or more smartphones and digital interventions have been proposed as a feasible means of disseminating health programs in homeless populations [21,22]. Thus, mHealth interventions have the potential to be universally accessible regardless of geographic, financial, and infrastructure constraints. mHealth platforms confer the advantages of appeal (self-pacing, instant availability, anonymity), reach, and cost-effectiveness [23-25]. This may be particularly important for meeting the needs of AYAs, who grew up in the digital age and are accustomed to interacting on smartphones and prefer internet-based self-help [24,26]. Smartphone adoption and internet use have skyrocketed among younger generations, with 95%-96% of teenagers and young adults in the United States owning a smartphone [27,28]. The internet serves as a primary means of physical- and mental health-related information-seeking and communication platforms for AYAs [26,29]. In addition, AYAs are reluctant to seek traditional forms of psychotherapy due to social stigma and discomfort in discussing personal problems and prefer digital interventions [24-26,30].

Despite the promise of mHealth interventions and the appeal to AYAs in particular, psychosocial mHealth interventions focused on improving QoL and psychosocial outcomes have not been evaluated or implemented among AYA oncology patients. This paper describes the protocol of an ongoing pilot RCT of a digitized version of PRISM deployed through a smartphone app (mobile app Promoting Resilience in Stress Management [mPRISM]).

## Methods

### Study Aims

The primary aim of this study is to evaluate the feasibility and acceptability of a self-guided mHealth resilience intervention (mPRISM) for AYAs with cancer. mPRISM content is based on stress and coping theory, resilience theory, and evidence-based psychosocial interventions. We hypothesize that mPRISM will be feasible as measured by ≥60% enrollment and ≥70% retention of participants to complete the study; and feasible, acceptable, and appropriate as measured by 3 brief well-established implementation measures (feasibility of intervention measure [FIM], acceptability of intervention measure [AIM], and intervention appropriateness measure [IAM] scores ≥4/5) [31]. Exploratory aims are to (1) examine preliminary efficacy assessed by validated patient-reported outcome measures at posttreatment and 3-month follow-up and (2) summarize digital usage metrics and engagement patterns under naturalistic conditions to inform future study design and to prepare participant instructions on how to use the app.

### Trial Design

**Figure 1:**
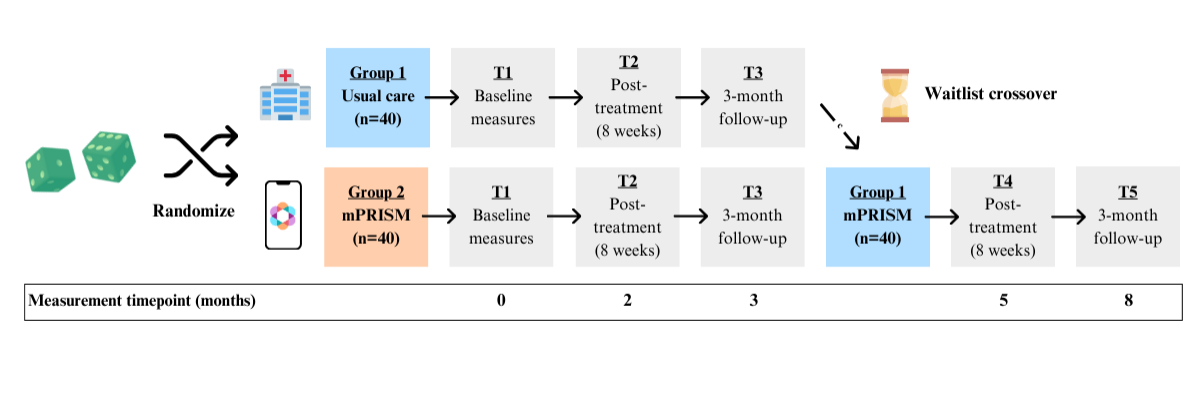
Schedule of all study activities. mPRISM: mobile app Promoting Resilience in Stress Management. T: timepoint.

This is a parallel, 2-arm, pilot RCT (Figure 1) [32]. Participants (N=80) with cancer will be enrolled and randomized to usual care (UC) or UC plus mPRISM at a single site. Using a waitlist design, mPRISM will be provided to the UC arm at a 3-month follow-up. Participant randomization to the study arm will occur after the completion of baseline surveys and in a 1:1 allocation ratio. As a waitlist design is being used, it is not possible to blind participants to the intervention condition [33]. Study outcomes, however, are completed independently by AYA participants to minimize bias.

### Participants

AYAs are eligible if they are (1) 12-25 years old; (2) within 12 months of diagnosis of new cancer; (3) receiving chemotherapy or radiation therapy at Seattle Children’s Hospital; (4) speak, read, or write English; and (5) have no cognitive limitations that would make participation difficult. We will provide iPads (Apple Inc) for the duration of the study for those who do not own a device.

Exclusion criteria include patient refusal to participate or parental refusal to participate for patients <18 years of age, patients who received a diagnosis of malignancy >12 months ago, patients with relapsed or refractory cancer, patients who have not received any chemotherapy or radiation therapy as part of their cancer treatment, cognitively or physically unable to participate, and previous participation in other PRISM-based studies. Clinical research coordinators will identify potential participants and prescreen through clinic rosters and electronic medical records. Recruitment by clinical research coordinators will occur remotely by phone, video, or SMS text messaging (ie, Zoom [Qumu Corp], Microsoft Teams, Webex [Cisco Systems], Skype [Microsoft Corp], email, SMS text messaging, and MyChart message [Epic Systems]).

### Intervention Conditions

#### UC

UC consists of standard nondirected supportive care provided for all patients including an assigned social worker who provides ad hoc services (mental health, financial, and housing support) at the request of the patient or family or medical team throughout cancer treatment. The AYA or their medical team can request referrals to psychology, psychiatry, pain medicine, child life, chaplaincy, palliative care, and art or music therapy.

#### Usual Care Plus mPRISM

**Figure 2:**
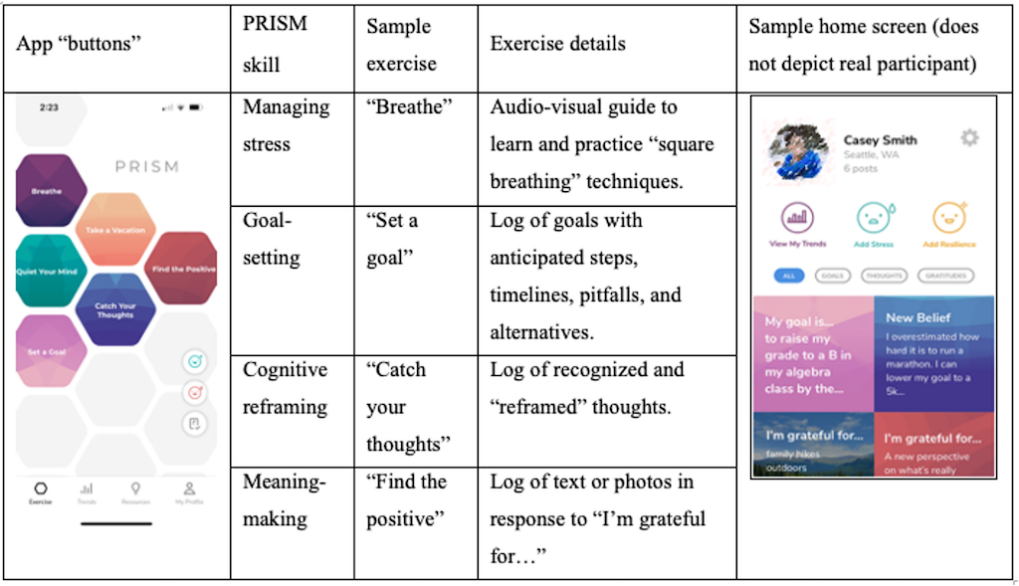
Selected examples of skills and practice materials on the mobile app version of Promoting Resilience in Stress Management. PRISM: Promoting Resilience in Stress Management.

In addition to UC, participants will receive access to mPRISM. PRISM is a brief, evidence-based resilience coaching intervention consisting of 4 scripted sessions delivered 1:1 by layperson coaches with high fidelity [11,14]. Sessions cover 4 core skills that are stress management (deep breathing, relaxation, and mindfulness techniques), goal setting (setting goals that are specific, measurable, actionable, and realistic, and planning around potential obstacles and barriers), cognitive reframing (identifying, challenging, and reframing negative thoughts), and meaning-making (identifying benefits, purpose, and meaning from cancer experience). Content from the in-person PRISM intervention was adapted to a mobile app (mPRISM), retaining core skills of the PRISM program (stress management, goal setting, cognitive reframing, and meaning-making [Figure 2]). Like the in-person PRISM program, mPRISM was co-designed with AYAs and with multiple iterations of stakeholder feedback. mPRISM contains tutorials showcasing audio-visual skills, dynamic skills practice opportunities, tracking systems of self-logged stress and resilience meters, a photo journal to review practiced skills, app notification reminders, and media links to other resources. AYAs randomized to mPRISM will receive a unique download code for the app. AYAs randomized to UC will receive a download code for the app after completion of their 3-month follow-up surveys. Once they receive access to mPRISM, all participants are instructed to use the app for 8 weeks.

#### Measures

Enrolled AYA participants will complete surveys at baseline before randomization, posttreatment (at 8 weeks), and 3-month follow-up (at 20 weeks). All surveys will be completed electronically by REDCap (Research Electronic Data Capture; Vanderbilt University) SMS text messaging or email survey invitation [34,35]. Survey responses will be scored based on measure scoring guidelines from the measure developers. In a waitlist design, the UC arm will receive mPRISM upon completion of the 3-month follow-up surveys to increase retention and optimize the use of the full sample size for analysis. This second wave of data collection for UC will include 2 additional measurement points at immediate posttreatment and 3 months later. Measure batteries at each timepoint take approximately 15-25 minutes to complete.

#### Data Storage

Data will be stored within a REDCap database on a secure, internal, HIPAA (Health Insurance Portability and Accountability Act)-compliant, password-protected Seattle Children’s server. Electronic consent will be stored in the same password-protected, secure database using the REDCap eConsent framework that securely collects, stores, and archives electronic consent information [34,35]. All electronic access to REDCap is encrypted.

#### Primary Outcomes: Feasibility, Acceptability, and Appropriateness

Feasibility is defined as ≥60% enrollment and ≥70% retention who complete the study. The FIM, AIM, and IAM are well-validated 4-item implementation science measures with strong reliability and validity [31]. Measures query perceptions of the feasibility, acceptability, and appropriateness of adopting an evidence-based practice. Items are rated on a 5-point Likert scale from “completely disagree” to “completely agree,” and total scores range from 1 to 5. Higher scores indicate greater acceptability, appropriateness, and feasibility, respectively. These 3 measures have been used independently and in conjunction. Feasibility, acceptability, and appropriateness cut-off scores are defined as measure scores ≥4 (on a 5-point scale).

#### Secondary and Exploratory Outcomes

##### Usability

The System Usability Scale (SUS) is a well-validated 10-item scale to evaluate the perceived usability of digital interventions with strong reliability and adequate validity [36]. Items are rated on a 5-point Likert scale for a total score ranging from 0 to 100, and scores ≥70 constitute adequate usability.

##### Health-Related Quality of Life

The PedsQL (Pediatric Quality of Life Inventory) 4.0 Generic and 3.0 Cancer Module include 50 items evaluating the HRQoL of AYAs with cancer and have strong reliability and validity [37]. Queries assess physical, emotional, social, and school well-being, plus cancer-related pain and hurt, nausea, procedural anxiety, treatment anxiety, worry, cognitive problems, perceived physical appearance, and communication. Items are rated on a 5-point Likert scale, and we will use the total scale score. Scores are linearly transformed to a 0 to 100 scale, with higher scores indicating better HRQoL.

##### Resilience

The Connor-Davidson Resilience Scale (CD-RISC) is a well-validated and widely used 10-item instrument to measure inherent resiliency that has demonstrated strong reliability and validity [38]. Questions revolve around personal problem-solving and approaches to adversity. Items are rated on a 5-point Likert scale; scores range from 0 to 40, and higher scores indicate higher resilience.

##### Distress

The Kessler Psychological Distress Scale (K6) is a 6-item scale that measures the level of psychological distress and has strong reliability and validity [39]. The instrument strongly discriminates between community cases and noncases of the *DSM-5* (*Diagnostic and Statistical Manual of Mental Disorders* [Fifth Edition]) psychiatric disorders such as serious emotional distress or serious mental illness. Items are rated on a 5-point Likert scale, scores range from 0 to 24, and higher scores indicate higher distress.

##### Anxiety and Depression

The Hospital Anxiety and Depression Scale (HADS) is a 7-item scale that assesses symptoms of anxiety and depression, respectively, in patients with serious illness [40]. It has been validated in AYAs with chronic illness and cancer survivors and has strong reliability and validity [41,42]. Items are scored 0-3 (subscale range 0-21), with scores ≥8 categorized as borderline abnormal, and ≥11 categorized as abnormal.

##### Digital Usage Metrics

Individual backend user data for mPRISM are collected in real time using Mixpanel, an event analytics service that tracks user engagement with mobile apps [43].

### Data Analysis

#### Power

The role of rigorous pilot studies such as this study is designed to determine feasibility [44,45]. Although not powered for efficacy, we will examine preliminary efficacy data for meaningful trends in terms of clinical significance with minimal clinically important difference (MCID) defined as half the SD for outcomes [46].

#### Primary Outcomes

Outcome domains will be assessed with Proctor’s framework [47] to determine implementation success (Table 1). Penetration will be assessed by clinic recruitment rates with ≥60% enrollment considered feasible. Uptake will be assessed through retention rates with ≥70% of participants completing the study considered feasible. We will apply top-box scoring [48] for the implementation measures, which is widely used in satisfaction research. Acceptability, appropriateness, and feasibility will be considered high if corresponding top-box scores are 80%.

**Table 1:** Proctor’s domains assessed in data analysis plan.

Domain	Data analysis plan
Penetration	Recruitment rate
Uptake	Retention rate
Feasibility, acceptability, and appropriateness	Top-box scoring of validated implementation measures
Patient outcomes	Regression models of validated function and symptomatology measures

#### Secondary and Exploratory Outcomes

Data from all participants who complete baseline surveys will be included in intent-to-treat analyses [49]. Before UC crosses over to receive mPRISM, the first phase of the study can be viewed as a parallel 2-armed RCT. Given the first phase parallel group RCT design, we will first compare posttreatment efficacy outcomes between mPRISM and UC using 2-sample independent *t* tests [50]. We will explore a range of effect sizes around what is observed in this pilot trial and assume larger SEs. Exploratory efficacy will be examined using the first phase parallel RCT data with regression models [44,45]. The effect of assessment time and other unbalanced confounders will be assessed as covariates in the models [51]. Potential covariates include age, gender, and diagnosis, which have been shown to influence psychosocial outcomes [52]. Estimation will be based on restricted maximum likelihood and significance based on the Wald *t* test [53]. With regard to digital usage data, we will summarize individual digital usage metrics (eg, number of usage sessions and total duration of use) using descriptive statistics and graphical tools.

### Ethical Considerations

All procedures are approved by the Seattle Children’s Hospital Institutional Review Board (STUDY00004006). The trial is registered at ClinicalTrials.gov (NCT05842902). All participants will provide informed consent (18-25 years old) or assent with parental consent (12-17 years old). Study participation is completely voluntary and participants are allowed to opt out of the study at any time. Privacy and confidentiality protections will be in accordance with institutional ethical guidelines. Study data are collected on secure, internal, HIPAA-compliant REDCap servers only accessible to the study team [34,35]. The collection of identifiable information is limited to that required to contact participants for study procedures and to report on sample demographics. Participants consented to use of data for study publications and no individually identifiable information will be reported in study publications. Participants will be compensated US $25 in electronic gift cards for completing each survey.

## Results

This project was funded in September 2022, and received IRB approval in November 2022. We began study enrollment in September 2023. As of July 2024, we have enrolled 25 participants and recruitment is ongoing. We expect to complete data collection in 2027.

## Discussion

### Principal Findings

The World Health Organization states that digital and mHealth technologies have the potential to revolutionize health care and improve access to services [54]. mHealth interventions provide (1) in-the-moment support in patients’ day-to-day lives, (2) have been proven to be cost-effective due to reduced personnel and infrastructure requirements [55], and (3) have the ability to reach patients in remote areas and those traditionally underserved. Although there are 325,000 health-related apps available for download as of 2017 and a growth rate of 25% year-to-year [56], there are no evidence-based psychosocial mHealth interventions for AYAs with cancer. This pilot RCT is designed to evaluate the feasibility and acceptability of the mPRISM intervention, a skills-based mobile app adapted from the in-person PRISM program. This ongoing trial takes a critical next step in translating evidence-based interventions to digital platforms, expanding the reach of evidence-based supportive care to the vulnerable populations who need them. We anticipate that mPRISM will be feasible, acceptable, and appropriate. Exploratory patient-reported outcomes will inform sample size for future powered trials. Individual backend user data will explore patterns of engagement with skills modules. We expect findings from this pilot trial to inform the testing of mPRISM in a larger scale multisite hybrid effectiveness-implementation RCT powered for efficacy outcomes.

### Limitations

This study has several anticipated challenges. First, this is a single-site pilot trial, which may limit the generalizability of findings. However, the strengths of this single-site study are established relationships with the clinic to ensure project completion and preliminary data for future scale-up initiatives. Second, it is possible that we may have higher attrition rates and lower adherence, which is common in smartphone-based mental health interventions [57]. However, members of our research team have over a decade of experience and background with digital behavioral health interventions in research settings that have demonstrated low attrition and high adherence rates. We will monitor attrition rates on a monthly basis. If attrition is >50%, we will increase the level of study coordinator contact to ensure project completion. This will provide information on potential behavioral nudge strategies to increase engagement and sustainability. Third, we are primarily using remote recruitment methods, which may lead to higher or lower recruitment rates than expected. Fourth, we do not provide instructions to participants on how to engage with mPRISM, although a recommended order of module completion is available on the home screen based on the in-person version of the intervention. Digital usage metrics will inform future study design and the potential development of more structured instructions on how best to engage with mPRISM. Fifth, participants are not blinded to intervention conditions, which is a potential source of bias. Here, we evaluate the additive value of mPRISM to UC which is the standard of care all patients receive. Sixth, this is a feasibility waitlist control trial design, which limits our ability to make causal inferences regarding efficacy. Nonetheless, we will examine exploratory trends of symptom change. Future larger-scale studies will incorporate active treatment comparison conditions (eg, another behavioral health app) and be sufficiently powered for efficacy. In addition, future studies will test and refine implementation strategies in multisite clinical settings and include adaptations for other languages, cultures, and patient groups.

### Conclusions

AYAs with cancer are a vulnerable population with unmet psychosocial needs. mHealth initiatives have the unique potential to scale up interventions and overcome structural barriers and unequal access to psychosocial treatment. Although our previous work suggests AYAs with cancer are interested in mHealth psychosocial interventions to improve QoL, resilience, and distress [58,59], such interventions have not yet been sufficiently evaluated or implemented among AYA oncology patients. mPRISM may serve as a potential mHealth intervention to fill this gap. This pilot trial will inform the necessary sample sizes and power of a future larger multisite hybrid effectiveness-implementation trial. Future studies will assess barriers and facilitators to mHealth psychosocial intervention adoption and maintenance, and strategies to increase and maintain mobile app engagement. This pilot study and future studies building on this work aim to expand the reach, adoption, and sustainability of evidence-based mHealth psychosocial interventions. Since the COVID-19 pandemic, digitally deployed supportive care has rapidly evolved and accelerated in research and practice settings [60]. This study has important clinical implications in the use of mHealth for psychosocial symptom self-management, exploring the potential integration of digital tools with psychosocial standard of care practices, and increasing access to care for underresourced groups.

## Appendix

Multimedia Appendix 1 External peer review report.

## Abbreviations

AIM: Acceptability of Intervention Measure
AYA: adolescent and young adult
CD-RISC: Connor-Davidson Resilience Scale
DSM-5: Diagnostic and Statistical Manual of Mental Disorders (Fifth Edition)
FIM: Feasibility of Intervention Measure
HADS: Hospital Anxiety and Depression Scale
HIPAA: Health Insurance Portability and Accountability Act
HRQoL: health-related quality of life
IAM: Intervention Appropriateness Measure
K6: Kessler Psychological Distress Scale
MCID: minimal clinically important difference
mHealth: mobile health
mPRISM: mobile app Promoting Resilience in Stress Management
PedsQL: Pediatric Quality of Life Inventory
PRISM: Promoting Resilience in Stress Management
QoL: quality of life
RCT: randomized controlled trial
REDCap: Research Electronic Data Capture
SUS: System Usability Scale
UC: usual care

## Data Availability

Deidentified human participants’ data from this research study will be banked indefinitely. The data sets generated during and analyzed during this study are not publicly available due to research involving minors at a single pediatric oncology treatment center and a geographically specific population but data are available from the corresponding author on reasonable request.
