# Supplementary material for: Mobile App Promoting Resilience in Stress Management for Adolescents and Young Adults With Cancer: Protocol for a Pilot Randomized Controlled Trial
Source: JMIR Res Protoc. 2024 Jul 30;13:e57950. doi: 10.2196/57950 (PMC11322713; doi:10.2196/57950)
Supplement: Multimedia Appendix 1 [file resprot_v13i1e57950_app1.pdf]

RESUME AND SUMMARY OF DISCUSSION: In this NCI Mentored Clinical Scientist Research Career Development Award (K08) resubmitted application, the Principal Investigator (PI), Dr. Lau proposes to evaluate feasibility, acceptability, and early efficacy of a mobile Health (mHealth) intervention, mobile Promoting Resilience in Stress Management (mPRISM) for Adolescents and Young Adults (AYAs) with cancer. Dr. Lau is an exceptionally strong, mentor supported, well trained and industrious clinical psychologist candidate with strong research training in clinical psychology, cancer survivorship including palliative care research, and mHealth intervention which are very relevant to this application. She possesses a very strong publication record in the field of effectiveness of interventions affecting young adult mental health, with the most recent work showing a strong commitment to cancer research. Dr. Lau has received institutional training grants, and she is supported by strong reference letters testifying her high enthusiasm, commitment and high potential for becoming an independent investigator. An improved and responsive career development plan (CDP) with career goal to establish an interdisciplinary psycho-oncology research program focused on the implementation of new and innovative psychosocial treatments for AYAs with cancer by leveraging digital technologies is described. The CDP is well aligned with the research plan, and additional details are now provided on training plan, scholarship, and mentorship structure. She has proposed training activities in areas of advanced training in clinical trials research, evaluation of mHealth interventions and to optimize engagement and adoption, advanced training in implementation science (IS) and research methods which are complementary and logical extension of her prior training and experience. The proposed training activities are adequate which includes appropriate course works, regular interactions with mentors, well planned timeline, benchmarks, and full evaluation of her research and career progress by mentors twice in a year. Research plan has strong clinical relevance, it is a logical extension of her mentor's intervention that has been well studied and broadly adapted, thus supporting its strong scientific premise and feasibility. Research plan is improved by addressing clarification of implementation science frameworks, and inclusion of interviews with targeted cohorts to identify barriers/facilitators. Research plan is a good fit for the candidate's experience and prior training as a clinical psychologist and work to develop the behavioral mobile health version of the intervention which gives a foundation for achieving the project's research goals. Proposed research is overall scientifically rigorous, includes validated measures of acceptability and strong mobile analytics, expanded to Spanish-speaking patients, and overall robust in research design allowing assessments of both facilitators and barriers to adherence to the intervention. Dr. Lau has assembled an outstanding and well-published mentoring and advisory team with complementary expertise in pediatric and AYA oncology, PRISM, mHealth for mental health, mobile analytics, clinical trials in medically ill populations, implementation science, palliative/supportive care, ethical research conduct in pediatric digital intervention science, statistics, qualitative research methods and adolescent mental health. Primary mentor is a leader in intervention trials focused on young adults with cancer, well-funded and possesses strong mentoring record in advancing trainees to research independence. Mentorship team is highly supportive and offers significant effort for training the mentee in proposed content areas and career development. Proposed research and career development of the candidate will occur in an outstanding environment of the University of Washington and the Seattle Children's Hospital, and 75% protected time to the candidate is promised. Dr. Lau is adequately responsive to the concerns of initial submission and the resubmission is significantly improved. Few remaining weaknesses, inadequate plan for differentiation from primary mentor to an independent line of research, a missed opportunity of comparing mPRISM to in-person PRISM, and inadequately strong support from the institute non contingent to this award were discussed. Strength outweighs weaknesses, and this improved resubmission is expected to deliver a high impact on the career development of the candidate as an independent investigator of psycho-oncology.

DESCRIPTION (provided by applicant): In addition to coping with their medical illness, Adolescents and Young Adults (AYAs) with cancer struggle with the age-related developmental challenges of

independence, education, employment, relationships, and identity. AYAs with cancer are at high risk of poor short-term and long-term outcomes, including poor mental health, social isolation, risky health behaviors, unemployment, and poor quality of life. With the rapid rise in digital technologies, skills-based psychosocial interventions are increasingly deployed via mobile health (mHealth) platforms. Such interventions show promising treatment effects, help overcome barriers to traditional in-person delivery models and are highly appealing to AYAs. However, mHealth interventions focused on improving quality of life and psychosocial outcomes have not yet been successfully evaluated or implemented among AYA cancer patients. This application builds on my prior work with the "Promoting Resilience in Stress Management" (PRISM) intervention for AYAs with cancer. This novel, brief, evidence-based 1:1 intervention teaches stress management, goal-setting, meaning making, cognitive-behavioral, and mindfulness strategies. I and my team have adapted this in-person intervention to a self-guided mHealth intervention, mPRISM. This mixed-methods proposal has the following 3 complementary aims: (Aim 1) To evaluate the feasibility, acceptability, and exploratory efficacy of mPRISM for AYAs with cancer in a pilot randomized trial; (Aim 2) To analyze backend digital usage patterns in order to identify and target aspects of digital engagement associated with improvement in psychosocial outcomes; and, (Aim 3) To assess barriers and facilitators to the adoption of mHealth interventions in clinical settings via semi-structured exit interviews with patient and clinic stakeholders. This proposal has technological, methodological, and dissemination innovations in potential for scale-up of interventions to overcome structural barriers to psychosocial treatment access. This K08 will provide opportunities to acquire skills and knowledge in: advanced clinical trials research as PI; mHealth research including intervention testing, digital analytics, and strategies to optimize engagement and adoption; and advanced implementation science methods. The research and career development plan, supported by a multi- disciplinary team of experts (in AYA oncology, implementation science, mHealth and mobile analytics, clinical trials research, advanced statistics, and qualitative methods) and in a rich academic environment, will support the PI's transition to an independent clinical-scientist who possesses the skills and expertise to use cutting- edge mobile health methods to promote the implementation of evidence-based care for AYA oncology patients. This proposal will inform the design of the PI's large-scale multi-site hybrid effectiveness-implementation trial of mPRISM (R01 proposal). Aligned with the NCI's priorities, this program of research will promote evidence- based behavioral interventions that improve quality of life, the utilization of portable low-cost technologies to expand access to care, and implementation science initiatives to bridge the research-practice gap.

PUBLIC HEALTH RELEVANCE: Cancer among Adolescents and Young Adults (AYAs) is associated with poor psychosocial outcomes and quality of life, and patients experience disproportionately high levels of anxiety, depression, and posttraumatic stress in relation to their younger pediatric and older adult counterparts. Findings from the proposed project will help address the unmet psychosocial needs of AYA cancer patients using a novel mobile health psychosocial care delivery model with the advantages of appeal, reach, and cost-effectiveness. Ultimately, this work may help improve patient health-related quality of life and emotional well-being, and reduce the burden of cancer, by expanding access to evidence-based care.

CRITIQUE: The written critiques of individual reviewers are provided in essentially unedited form in this section. Please note that critiques and criteria scores, prepared prior to the review meeting, may not have been revised following discussions at the meeting. The "Resume and Summary of Discussion" section summarizes the final opinions of the review committee.

CRITIQUE 1

Candidate:	1
Career Development Plan/Career Goals /Plan to Provide Mentoring:	3

Research Plan: 2
Mentor(s), Co-Mentor(s), Consultant(s), Collaborator(s): 2
Environment Commitment to the Candidate: 3

Overall Impact: Dr. Lau is an Acting Assistant Professor in the University of Washington School of Medicine Department of Psychiatry & Behavioral Sciences and a UW K12 Implementation Science Scholar. She received her Ph.D. in Clinical Psychology from Harvard University under the mentorship of Dr. John Weisz and subsequently completed a T32 research fellowship at UW in palliative care research. Dr. Lau proposes a mixed-methods project with complementary aims that will evaluate feasibility, acceptability, and early efficacy of mPRISM for AYAs with cancer as well as examining both backend digital usage patterns and barriers/facilitators to the adoption of mHealth interventions. Her resubmitted application has resulted in a tremendous response to initial reviewer's concerns. Moderate concerns remain for this current Reviewer about the Institution's commitment to this candidate and Dr. Lau's freedom to individuate from her primary mentor, given that the PRISM intervention in central to her line of research.

1. Candidate:
Strengths
- All initial reviewers rated this candidate "1."
- Strong letter informing candidate's likelihood for a long career as an independent researcher.
- Strong publication record (>20 published; >9 first authored).

Weaknesses
- None noted.

2. Career Development Plan/Career Goals & Objectives:
Strengths
- Adequate response to initial Reviewer concerns, including those re: synchronicity of training plan with research strategy and encouragement to increase training in implementation science.
- Additional details are now provided on training plan, scholarship, and mentorship structure.
- Logical extension of Dr. Rosenberg's PRISM to an mHealth platform.

Weaknesses
- Concerns from this reviewer are around how she will differentiate her from her mentorship team with regard to an independent line of research.

3. Research Plan:
Strengths
- Candidate provided adequate responses to initial Reviewer comments, ranging from scores of 3-6, regarding Research Plan. This included the candidate addressing clarification of implementation science frameworks, justification for comparing mPRISM to usual care, and inclusion of interviews with targeted cohorts to identify barriers/facilitators.
- mHealth initiatives are important and necessary within AYA cancer populations, given that they are much more likely to engage with psychosocial care in a virtual format than via in-person modalities.
- Inclusion of mobile analytics is strong.

Weaknesses
- Concern about candidate's ability to differentiate their own line of ePRISM research from that of Dr. Rosenberg's primary line.
- Preliminary research funded by donor funding, with no evidence of candidate's successful competition for intra/extramural funding.

4. Mentor(s), Co-Mentor(s), Consultant(s), Collaborator(s):
Strengths
- Dr. Rosenberg is primary mentor and has successfully mentored multiple junior faculty through acquisition and carry-through of NIH projects.
- Co-Mentors Drs. Ben-Zeev, J. Randall Curtis, and Bryan Weiner provide necessary expertise in mHealth, clinical trials in medically ill populations, and implementation science, respectively.

Weaknesses
- Minor concerns remain regarding how additional training for the candidate will be beneficial.

5. Environment and Institutional Commitment to the Candidate:
Strengths
- The Department of Psychiatry and Behavioral Sciences within the University of Washington and the Seattle Children's Research Institute provide extraordinary training environments for the candidate to carry out CDA activities.

Weaknesses
- Candidate is an "Acting Assistant Professor" since 2019. While this may be standard at UW, it remains unclear how this will impact her future integration into a faculty role if this award were to be funded.

Protections for Human Subjects: Acceptable Risks and Adequate Protections
- Distribution justified scientifically.

Data and Safety Monitoring Plan (Applicable for Clinical Trials Only): Acceptable

Inclusion Plans
- Sex/Gender: Distribution justified scientifically
- Race/Ethnicity: Distribution justified scientifically
- For NIH-Defined Phase III trials, Plans for valid design and analysis: Not applicable
- Inclusion/Exclusion Based on Age: Distribution justified scientifically
  - Sex/Gender, Race/Ethnicity, and Age Distributions are justified.

Vertebrate Animals: Not Applicable (No Vertebrate Animals)

Biohazards: Not Applicable (No Biohazards)

Training in the Responsible Conduct of Research: Acceptable
Comments on Format (Required):
- CITI training is acceptable.

Comments on Subject Matter (Required):
- RCR for NIH and SBM clinical trials is appropriate.

Comments on Faculty Participation (Required; not applicable for mid- and senior-career awards):
- Appropriate.

Comments on Duration (Required):
- Appropriate.

Comments on Frequency (Required):
- Appropriate.

Resource Sharing Plans: Acceptable

Authentication of Key Biological and/or Chemical Resources: Not Applicable (No Relevant Resources)

Budget and Period of Support: Recommend as Requested

CRITIQUE 2

Candidate:	1
Career Development Plan/Career Goals /Plan to Provide Mentoring:	2
Research Plan:	4
Mentor(s), Co-Mentor(s), Consultant(s), Collaborator(s):	1
Environment Commitment to the Candidate:	2

Overall Impact: This is a resubmission of a K08 application that received an impact score of 41 in 2021. Dr. Lau has been an Acting Assistant Professor at University of Washington in Psychiatry and Behavioral Sciences since 2019. She states her career goal is to establish an interdisciplinary psycho-oncology research program focused on the implementation of new and innovative psychosocial treatments for AYAs with cancer by leveraging digital technologies. She has a strong publication record. She is currently funded on a K12 and T32 but has no prior independent funding.

There are many strengths to this application, including a strong candidate, established mentors, and the leveraging of her mentor's research program to facilitate this application.

The career development plan proposes training activities in the following areas: (1) Advanced training in clinical trials research as principal investigator, (2) Training to evaluate mHealth interventions and optimize engagement and adoption, and (3) Advanced training in implementation science (IS) research methods.

The research plan aims include: (1) Evaluate the feasibility and acceptability of mPRISM for AYAs with cancer in a pilot trial, (2) Evaluate patterns of AYA engagement and mPRISM treatment dose via mobile analytics, and (3) Assess barriers and facilitators to adopting mHealth interventions in "real-world" settings. While not particularly novel, this is a logical extension of her mentor's intervention that has been well studied and broadly adapted.

This is a feasible research project with a solid career development plan. There remains some concern about how the candidate will develop her own independent research program given how entirely reliant this research is on her mentor's intervention.

1. Candidate:
Strengths
- Dr. Lau received a PhD in Clinical Psychology at Harvard in 2016. She completed residency in Clinical Psychology in 2016 and postdoctoral fellowship in pediatric palliative care in 2019, both at University of Washington. She has been an Acting Assistant Professor at University of Washington in Psychiatry and Behavioral Sciences since 2019.
- She has a strong publication record, with 26 including 12 first-authored publications.
- Currently funded on a K12 and T32.

Weaknesses
- No funding history.

2. Career Development Plan/Career Goals & Objectives:

Strengths

- Dr. Lau proposes a career development plan including: (1) Advanced training in clinical trials research as principal investigator, (2) Training to evaluate mHealth interventions and optimize engagement and adoption, and (3) Advanced training in implementation science (IS) research methods.
- The goals and activities outlined are appropriate to prepare this candidate for an independent research career.

Weaknesses

- None noted.

3. Research Plan

Strengths

- The following aims are proposed: (1) Evaluate the feasibility and acceptability of mPRISM for AYAs with cancer in a pilot trial, (2) Evaluate patterns of AYA engagement and mPRISM treatment dose via mobile analytics, and (3) Assess barriers and facilitators to adopting mHealth interventions in "real-world" settings.
- The proposed research builds upon her mentor's existing intervention that has been well studied and has generated many extended applications (e.g., diabetes). The applicant has been working on the mPRISM app development since 2017.
- The candidate has expanded the proposed study to include Spanish-speaking patients.
- The research topic is clinically relevant and a good fit for the candidate's experience and training.

Weaknesses

- The rationale, in response to reviewer concerns, for comparing mPRISM to "usual care" rather than to in-person PRISM was not compelling. Since the candidate is already leveraging her mentor's ongoing PRISM studies, this seems like an obvious missed opportunity to do a more meaningful study. To say that PRISM is not yet standard of care is really not a convincing reason not to compare to mPRISM considering that PRISM is such a heavily studied intervention.
- Adaptation of the intervention to mobile delivery is not particularly novel.
- The application is dense, which makes it harder to pull out key design elements.

4. Mentor(s), Co-Mentor(s), Consultant(s), Collaborator(s):

Strengths

- Mentors are well established in this area of study.
- Primary Mentor: Abby Rosenberg, MD (pediatric and AYA oncology, PRISM creator); Associate Professor at UW, Director of the Palliative Care and Resilience Research (PCAR) laboratory at the Seattle Children's Research Institute.
- Co-Mentor: Bryan Weiner, PhD (implementation science), Professor at UW Departments of Global Health and Health Services.
- Co-Mentor: Dror Ben-Zeev, PhD (mHealth for mental health, mobile analytics); Professor in the UW Department of Psychiatry and Behavioral Sciences, Director of the Behavioral Research in Technology and Engineering (BRiTE) Center.
- Co-Mentor: Randall Curtis, MD, MPH (clinical trials, palliative/supportive care); Professor at UW Department of Medicine, Director of the Cambia Palliative Care Center of Excellence.
- Advisors include: Chuan Zhou, PhD (statistics and analyses), Krysta Barton, PhD (qualitative research methods), and Tonya Palermo, PhD (ethical research conduct in pediatric digital intervention science); Elizabeth McCauley, PhD (adolescent mental health).

Weaknesses

- None noted.

5. Environment and Institutional Commitment to the Candidate:
Strengths
- The department chair assures 75% time dedicated to this application if awarded.
- This institution provides an exceptional environment for the candidate's research development.

Weaknesses
- None noted.

Protections for Human Subjects: Acceptable Risks and Adequate Protections
- Risks and protections are detailed in the application. They are acceptable.

Data and Safety Monitoring Plan (Applicable for Clinical Trials Only): Acceptable
- A data safety monitoring plan is established. Details were provided for monitoring and reporting.

Inclusion Plans
- Sex/Gender: Distribution justified scientifically
- Race/Ethnicity: Distribution justified scientifically
- For NIH-Defined Phase III trials, Plans for valid design and analysis: Not applicable
- Inclusion/Exclusion Based on Age: Distribution justified scientifically
  - Race/ethnicity/gender/age inclusion is representative of the population being sampled.

Vertebrate Animals: Not Applicable (No Vertebrate Animals)

Biohazards: Not Applicable (No Biohazards)

Resubmission: The candidate was responsive to some but not all of the prior reviews. The candidate is advised to mark changes in a resubmission to assist with the review.

Training in the Responsible Conduct of Research: Acceptable
Comments on Format (Required):
- Mentoring, coursework, online

Comments on Subject Matter (Required):
- Ethics, human subject's protection, authorship, COI, research misconduct, data acquisition

Comments on Faculty Participation (Required; not applicable for mid- and senior-career awards):
- Mentoring

Comments on Duration (Required):
- Ongoing, appropriate

Comments on Frequency (Required):
- ongoing mentoring, appropriate

Resource Sharing Plans: Acceptable

Budget and Period of Support: Recommend as Requested

CRITIQUE 3

Candidate:	1
Career Development Plan/Career Goals /Plan to Provide Mentoring:	1
Research Plan:	1

Mentor(s), Co-Mentor(s), Consultant(s), Collaborator(s): 1
Environment Commitment to the Candidate: 3

Overall Impact: This is a revised application from Dr. Lau that is highly responsive and thoughtful to address the prior critiques. Dr. Lau is a trained clinical psychologist who seeks additional skills for comparing their original mobile health intervention designed to improve quality of life outcomes for youth and young adult cancer survivors with usual source of care. An industrious scientist, the candidate has shown strong productivity in this area, and an independent trajectory from previous mentors with whom candidate has worked for the last four years by branching interventions into mobile health and gaining skills to do independent work in implementation science. The training plan complements the timeline of each of the study's aims. Mentors are experts in their respective content areas of cancer survivorship, implementation science, and mhealth interventions. The Department has supported the candidate's research career and shows a strong commitment to the candidate's long-term career and development. This candidate shows great potential to develop into an independent investigator, and the project is an important opportunity to bridge gaps in evaluation of an intervention that for which there is a clear plan for an R01-level intervention (comparison of eHealth version of intervention to in-person intervention).

1. Candidate:
Strengths
- Application builds on a mobile health intervention for young adult cancer survivors, previously developed by the candidate and funded by a K12.
- Candidate has published over 20 papers already on the effectiveness of interventions affecting young adult mental health, with the most recent work showing a commitment to cancer research.
- Reference letters show high enthusiasm and commitment to candidate.

Weaknesses
- None noted.

2. Career Development Plan/Career Goals & Objectives:
Strengths
- Progress will be monitored by a team of co-mentors meeting weekly or monthly, plus a full written evaluation twice per year.
- Candidate's prior training as a clinical psychologist and work to develop the behavioral mobile health version of the intervention give a foundation for achieving the project's research goals.
- Training plan includes advanced implementation science methods, which will allow the candidate to be able to launch their own studies after the completion of the K08.

Weaknesses
- None noted.

3. Research Plan:
Strengths
- Research design includes validated measures of acceptability and feasibility.
- Scientific premise is established by previously published work by candidate and mentor.
- Alignment between training plan and research skills is evidence in proposed coursework in longitudinal, multi-level analysis, and advanced skills in implementation science.
- Conducting interviews among people with varied levels of adherence to the intervention adds robustness to the research design, which can now assess both promoters and barriers to adherence.

Weaknesses

- None noted.

4. Mentor(s), Co-Mentor(s), Consultant(s), Collaborator(s):
Strengths
- Primary mentor is a leader in intervention trials focused on young adults with cancer, with several R01s, and is an experienced mentor who has had success advancing trainees in academia.
- Candidate's project is distinct from mentor's project by focusing on mobile health.
- Mentorship team offers sufficient effort for training mentee in proposed content areas.

Weaknesses
- None noted.

5. Environment and Institutional Commitment to the Candidate:
Strengths
- Department states a clear existing commitment to candidate and candidate and anticipates successful promotion of the candidate.
- Institution has a Children's Hospital, Children's Institute, and is attached to a top 10 pediatric Cancer center, all of which the candidate has access to and will enhance ability to complete the project.

Weaknesses
- Institutional letter still does not clearly state that the institution will support candidate, regardless of the outcome of the grant; currently, it states candidate will be supported through the award and beyond, but not despite awarding of the grant.

Protections for Human Subjects: Acceptable Risks and Adequate Protections
- Adequately addresses risk and ways team will minimize risk, as well as consenting processes for those above and below 18.
- Also assess what will happen if patients with high self-reports of distress are found by the study investigators.

Data and Safety Monitoring Plan (Applicable for Clinical Trials Only): Acceptable
- For a study of relatively low risks, having the PI and study team monitor safety is likely fine

Inclusion Plans
- Sex/Gender: Distribution justified scientifically
- Race/Ethnicity: Distribution justified scientifically
- For NIH-Defined Phase III trials, Plans for valid design and analysis: Not applicable
- Inclusion/Exclusion Based on Age: Distribution justified scientifically
  - Distributions justified by clinical population demographics

Vertebrate Animals: Not Applicable (No Vertebrate Animals)

Biohazards: Not Applicable (No Biohazards)

Resubmission: The revised application is highly responsive to prior critiques. The revised training and research plan have increased the robustness of the training opportunities, especially in implementation science, and increased the rigor of the analysis.

Training in the Responsible Conduct of Research: Acceptable
Comments on Format (Required):

- Face-to-face lectures, group discussions, and online modules

Comments on Subject Matter (Required):
- Protection of human subjects, data acquisition and management, peer review and responsible authorship, research misconduct, conflicts of interests, mentor/mentee responsibilities, collaborative science, and societal impacts of scientific research

Comments on Faculty Participation (Required; not applicable for mid- and senior-career awards):
- Some of the sessions are faculty-led

Comments on Duration (Required):
- At least 8+ hours per year

Comments on Frequency (Required):
- Annually

Resource Sharing Plans: Acceptable

Authentication of Key Biological and/or Chemical Resources: Not Applicable (No Relevant Resources)

Budget and Period of Support: Recommend as Requested

Recommended budget modifications or possible overlap identified: KL12 runs through August 2022, but generally institutional awards can easily be reduced once extramural funding becomes available

THE FOLLOWING SECTIONS WERE PREPARED BY THE SCIENTIFIC REVIEW OFFICER TO SUMMARIZE THE OUTCOME OF DISCUSSIONS OF THE REVIEW COMMITTEE, OR REVIEWERS' WRITTEN CRITIQUES, ON THE FOLLOWING ISSUES:

PROTECTION OF HUMAN SUBJECTS: ACCEPTABLE

INCLUSION OF WOMEN PLAN: ACCEPTABLE

INCLUSION OF MINORITIES PLAN: ACCEPTABLE

INCLUSION ACROSS THE LIFESPAN: ACCEPTABLE

COMMITTEE BUDGET RECOMMENDATIONS: The budget was recommended as requested.

Footnotes for 1 K08 CA263474-01A1; PI Name: Lau, Nancy

NIH has modified its policy regarding the receipt of resubmissions (amended applications).See Guide Notice NOT-OD-18-197 at https://grants.nih.gov/grants/guide/notice-files/NOT-OD-18-197.html. The impact/priority score is calculated after discussion of an application by averaging the overall scores (1-9) given by all voting reviewers on the committee and multiplying by 10. The criterion scores are submitted prior to the meeting by the individual reviewers assigned to an application, and are not discussed specifically at the review meeting or calculated into the overall impact score. Some applications also receive a percentile

ranking. For details on the review process, see http://grants.nih.gov/grants/peer_review_process.htm#scoring.

MEETING ROSTER

Career Development Study Section (J)
National Cancer Institute Initial Review Group
NATIONAL CANCER INSTITUTE
NCI-J
10/12/2021 - 10/13/2021

Notice of NIH Policy to All Applicants: Meeting rosters are provided for information purposes only. Applicant investigators and institutional officials must not communicate directly with study section members about an application before or after the review. Failure to observe this policy will create a serious breach of integrity in the peer review process, and may lead to actions outlined in NOT-OD-14-073 at https://grants.nih.gov/grants/guide/notice-files/NOT-OD-14-073.html, NOT-OD-15-106 at https://grants.nih.gov/grants/guide/notice-files/NOT-OD-15-106.html, and NOT-OD-18-115 at https://grants.nih.gov/grants/guide/notice-files/NOT-OD-18-115.html, including removal of the application from immediate review.

CHAIRPERSON(S)

DIXON, DAN ALAN, PHD
PROFESSOR DEPARTMENT OF MOLECULAR BIOSCIENCES
CO-LEADER, DRUG DISCOVERY, DELIVERY, AND EXPERIMENTAL THERAPEUTICS PROGRAM
UNIVERSITY OF KANSAS CANCER CENTER
UNIVERSITY OF KANSAS
LAWRENCE, KS 66045

EPPLEIN, MEIRA, PHD
ASSOCIATE PROFESSOR
DEPARTMENT OF POPULATION HEALTH SCIENCES
DUKE CANCER INSTITUTE
DUKE UNIVERSITY SCHOOL OF MEDICINE
DURHAM, NC 27710

MEMBERS

AGARWAL, RAJESH, PHD
PROFESSOR AND VICE CHAIRMAN DEPARTMENT OF PHARMACEUTICAL SCIENCES CO-LEADER, CANCER PREVENTION AND CONTROL PROGRAM
UNIVERSITY OF COLORADO CANCER CENTER
SKAGGS SCHOOL OF PHARMACY AND PHARMACEUTICAL SCIENCES
UNIVERSITY OF COLORADO
AURORA, CO 80045

BAKER, JUSTIN N, MD *
CHIEF, DIVISION OF PALLIATIVE CARE AND QUALITY OF LIFE
DEPARTMENT OF ONCOLOGY
ST. JUDE CHILDREN’S RESEARCH HOSPITAL
MEMPHIS, TN 38106

BANERJEE, IMON, PHD *
ASSOCIATE PROFESSOR
DEPARTMENT OF RADIOLOGY
ARIZONA STATE UNIVERSITY
SENIOR ASSOCIATE CONSULTANT
MAYO CLINIC
SCOTTSDALE, AZ 85259

BERNT, KATHRIN M, MD *
ASSISTANT PROFESSOR
DEPARTMENT OF PEDIATRIC ONCOLOGY
CHILDREN'S HOSPITAL OF PHILADELPHIA
UNIVERSITY OF PENNSYLVANIA
PHILADELPHIA, PA 19104

BHOWMICK, NEIL A., PHD
PROFESSOR OF MEDICINE
DEPARTMENT OF MEDICINE
DIRECTOR, CANCER BIOLOGY PROGRAM
SAMUEL OSCHIN COMPREHENSIVE CANCER INSTITUTE
CEDARS-SINAI MEDICAL CENTER
LOS ANGELES, CA 90048

BOCK, CATHRYN H, PHD *
ASSOCIATE PROFESSOR
DEPARTMENT OF ONCOLOGY
KARMANOS CANCER INSTITUTE
WAYNE STATE UNIVERSITY
DETROIT, MI 48201

CHEN, HERBERT, MD *
CHAIR OF THE DEPARTMENT OF SURGERY
DEPARTMENT OF SURGERY
UNIVERSITY OF ALABAMA SCHOOL OF MEDICINE
BIRMINGHAM, AL 35233

CHIAPPINELLI, KATHERINE B, PHD *
ASSISTANT PROFESSOR
DEPARTMENT OF MICROBIOLOGY,
IMMUNOLOGY AND TROPIC
GEORGE WASHINGTON UNIVERSITY
WASHINGTON, DC 20052

COSGROVE, VICTORIA EILEEN, PHD *
CLINICAL ASSOCIATE PROFESSOR
DIVISION OF CHILD AND ADOLESCENT PSYCHIATRY
SCHOOL OF MEDICINE
STANFORD UNIVERSITY
STANFORD, CA 94305

DAI, MUSHUI, PHD *
PROFESSOR
DEPARTMENT OF MOLECULAR AND MEDICAL GENETICS
CO-DIRECTOR OF GRADUATE STUDIES
SCHOOL OF MEDICINE
OREGON HEALTH & SCIENCES UNIVERSITY
PORTLAND, OR 97239

DEAN, LORRAINE TIERA, SCD
ASSOCIATE PROFESSOR DEPARTMENT OF EPIDEMIOLOGY
DEPARTMENT OF HEALTH POLICY AND MANAGEMENT
JOHNS HOPKINS BLOOMBERG SCHOOL OF PUBLIC HEALTH
SIDNEY KIMMEL CANCER CENTER
JOHNS HOPKINS UNIVERSITY SCHOOL OF MEDICINE
BALTIMORE, MD 21205

DENG, YIBIN, PHD, MD
PROFESSOR OF CANCER GENETICS
DEPARTMENT OF UROLOGY
THE UNIVERSITY OF MINNESOTA
MEDICAL SCHOOL
MINNEAPOLIS, MN 55455

DUDLEY, ANDREW C., PHD
ASSOCIATE PROFESSOR
DEPARTMENT OF MICROBIOLOGY, IMMUNOLOGY AND
CANCER BIOLOGY
UNIVERSITY OF VIRGINIA
CHARLOTTESVILLE, VA 22908

GANEM, NEIL J., PHD
ASSOCIATE PROFESSOR
DIVISION OF HEMATOLOGY AND ONCOLOGY
DEPARTMENT OF PHARMACOLOGY AND EXPERIMENTAL
THERAPEUTICS
BOSTON UNIVERSITY SCHOOL OF MEDICINE
BOSTON, MA 02118

GIBBONS, DON LYNN, PHD, MD *
PROFESSOR
DEPARTMENT OF THORACIC
HEAD AND NECK MEDICAL ONCOLOGY
UNIVERSITY OF TEXAS MD ANDERSON CANCER CENTER
HOUSTON, TX 77030

JAIN, MANEESH, PHD
PROFESSOR
DEPARTMENT OF BIOCHEMISTRY AND MOLECULAR
BIOLOGY
COLLEGE OF MEDICINE
FRED AND PAMELA BUFFETT CANCER CENTER
UNIVERSITY OF NEBRASKA MEDICAL CENTER
OMAHA, NE 68198

KAHALLEY, LISA SCHUM, PHD
ASSOCIATE PROFESSOR
DEPARTMENT OF PEDIATRICS
SECTION OF PSYCHOLOGY
BAYLOR COLLEGE OF MEDICINE
TEXAS CHILDREN'S HOSPITAL
HOUSTON, TX 77030-2399

KROGSGAARD, MICHELLE, PHD
ASSOCIATE PROFESSOR
DEPARTMENT OF PATHOLOGY
PERLMUTTER CANCER CENTER
NEW YORK UNIVERSITY SCHOOL OF MEDICINE
NEW YORK, NY 10016

LO, HUI-WEN, PHD
PROFESSOR
DEPARTMENT OF CANCER BIOLOGY
ASSOCIATE DIRECTOR FOR BASIC SCIENCES
WAKE FOREST BAPTIST COMPREHENSIVE CANCER
CENTER
WAKE FOREST SCHOOL OF MEDICINE
WINSTON-SALEM, NC 27157

LOERZEL, VICTORIA, PHD *
PROFESSOR
BEAT M AND JILL L KAHLI ENDOWED PROFESSOR
COLLEGE OF NURSING
UNIVERSITY OF CENTRAL FLORIDA
ORLANDO, FL 32826

LOWE, DEVIN B, PHD *
ASSISTANT PROFESSOR
DEPARTMENT OF IMMUNOTHERAPEUTICS AND
BIOTECHNOLOGY
TTUHSC SCHOOL OF PHARMACY
ABILENE, TX 79601

MCGRADY, MEGHAN E., PHD
RESEARCH ASSOCIATE PROFESSOR OF PEDIATRICS
DIVISION OF BEHAVIORAL MEDICINE
AND CLINICAL PSYCHOLOGY
LEADER, YOUNG ADULT ONCOLOGY PSYCHOSOCIAL
PROGRAM
CINCINNATI CHILDREN’S HOSPITAL MEDICAL CENTER
CINCINNATI, OH 45229-3026

RINK, LORI, PHD
ASSISTANT PROFESSOR
MOLECULAR THERAPEUTICS PROGRAM
FOX CHASE CANCER CENTER
PHILADELPHIA, PA 19111

ROGERS, CHARLES R., MPH, PHD
ASSISTANT PROFESSOR
DIVISION OF PUBLIC HEALTH
DEPARTMENT OF FAMILY AND PREVENTIVE MEDICINE
UNIVERSITY OF UTAH SCHOOL OF MEDICINE
SALT LAKE CITY, UT 84108

ROMESSER, PAUL BERNARD, MD *
ASSISTANT ATTENDING
DEPARTMENT OF RADIATION ONCOLOGY
MEMORIAL SLOAN-KETTERING CANCER CENTER
NEW YORK, NY 10065

SETIAWAN, VERONICA WENDY, PHD
PROFESSOR
DEPARTMENT OF PREVENTIVE MEDICINE
KECK SCHOOL OF MEDICINE
NORRIS COMPREHENSIVE CANCER CENTER
UNIVERSITY OF SOUTHERN CALIFORNIA
LOS ANGELES, CA 90033

TANG, LI MD, PHD
ASSOCIATE MEMBER
DEPARTMENT OF CANCER PREVENTION AND CONTROL
ROSWELL PARK CANCER INSTITUTE
BUFFALO, NY 14263

TRAN, DAVID D. MD, PHD
ASSOCIATE PROFESSOR OF NEUROSURGERY,
NEUROLOGY AND MEDICINE CHIEF, DIVISION OF NEURO-ONCOLOGY
DEPARTMENT OF NEUROSURGERY
MCKNIGHT BRAIN INSTITUTE
THE UNIVERSITY OF FLORIDA COLLEGE OF MEDICINE AND
UNIVERSITY OF FLORIDA HEALTH CANCER CENTER
GAINESVILLE, FL 32610-0265

WAHL, DANIEL R, MD, PHD *
ASSISTANT PROFESSOR
DEPARTMENT OF RADIATION ONCOLOGY
UNIVERSITY OF MICHIGAN
ANN ARBOR, MI 48109

WIITA, ARUN P. MD, PHD
ASSOCIATE PROFESSOR IN RESIDENCE
DEPARTMENT OF LABORATORY MEDICINE
UNIVERSITY OF CALIFORNIA, SAN FRANCISCO
SAN FRANCISCO, CA 94107

WOYACH, JENNIFER A., MD
ASSOCIATE PROFESSOR OF MEDICINE
DEPARTMENT OF INTERNAL MEDICINE
THE OHIO STATE UNIVERSITY
COLUMBUS, OH 43210

ZHOU, GANG, PHD
ASSOCIATE PROFESSOR, CANCER IMMUNOLOGY,
INFLAMMATION AND TOLERANCE PROGRAM
GEORGIA CANCER CENTER
COLLEGE OF GRADUATE STUDIES
AUGUSTA UNIVERSITY
AUGUSTA , GA 30912

SCIENTIFIC REVIEW OFFICER

DEB, TUSHAR, PHD
SCIENTIFIC REVIEW OFFICER
RESOURCES & TRAINING REVIEW BRANCH
DIVISION OF EXTRAMURAL ACTIVITIES
NATIONAL CANCER INSTITUTE
NATIONAL INSTITUTES OF HEALTH
ROCKVILLE, MD 20850

EXTRAMURAL SUPPORT ASSISTANT

WILSON, BRIDGETTE
EXTRAMURAL SUPPORT ASSISTANT
RESOURCE & TRAINING REVIEW BRANCH
DIVISION OF EXTRAMURAL ACTIVITIES
NATIONAL CANCER INSTITUTE- SHADY GROVE
NATIONAL INSTITUTES OF HEALTH
BETHESDA, MD 20892

PROGRAM REPRESENTATIVE

BIAN, YANSONG MD, PHD
PROGRAM DIRECTOR, CANCER TRAINING BRANCH
CENTER FOR CANCER TRAINING
NATIONAL CANCER INSTITUTE
NATIONAL INSTITUTES OF HEALTH
ROCKVILLE, MD 20850

JAKOWLEW, SONIA B, PHD
PROGRAM DIRECTOR
CANCER TRAINING BRANCH
CENTER FOR CANCER TRAINING
NATIONAL CANCER INSTITUTE
NATIONAL INSTITUTES OF HEALTH
BETHESDA, MD 20892

LIM, SUSAN E, PHD
PROGRAM DIRECTOR
CANCER TRAINING BRANCH
CENTER FOR CANCER TRAINING
NATIONAL CANCER INSTITUTE
NATIONAL INSTITUTES OF HEALTH
ROCKVILLE, MD 20850

RADAEV, SERGEI, PHD
PROGRAM DIRECTOR
OFFICE OF CANCER CENTERS
NATIONAL CANCER INSTITUTE
NATIONAL INSTITUTE OF HEALTH
BETHESDA, MD 20892

* Temporary Member. For grant applications, temporary members may participate in the entire meeting or may review only selected applications as needed.

Consultants are required to absent themselves from the room during the review of any application if their presence would constitute or appear to constitute a conflict of interest.
